# Identification of novel mutations in FFPE lung adenocarcinomas using DEPArray sorting technology and next-generation sequencing

**DOI:** 10.1007/s13353-018-0439-4

**Published:** 2018-03-10

**Authors:** Ji Won Lee, Jong-Yeon Shin, Jeong-Sun Seo

**Affiliations:** 1 Gongwu Genomic Medicine Institute (G2MI), Medical Research Center, Seoul National University Bundang Hospital, Seongnamsi, 13605 Republic of Korea; 2 Genomic Medicine Institute (GMI), Medical Research Center, Seoul National University, Seoul, 03080 Republic of Korea; 3 Department of Biomedical Sciences, Seoul National University College of Medicine, Seoul, Republic of Korea; 4 Macrogen Genome Institute, Medical Research Center, Seoul National University Bundang Hospital, Seongnamsi, 13605 Republic of Korea

**Keywords:** Novel mutation, Heterogeneity of FFPE, Next-generation sequencing, Pure cell sorting

## Abstract

Formalin-fixed paraffin-embedded (FFPE) tissues are utilized as the standard diagnostic method in pathology laboratories. However, admixture of unwanted tissues and shortage of normal samples, which can be used to detect somatic mutation, are considered critical factors to accurately diagnose cancer. To explore these challenges, we sorted the pure tumor cells from 22 FFPE lung adenocarcinoma tissues via Di-Electro-Phoretic Array (DEPArray) technology, a new cell sorting technology, and analyzed the variants with next-generation sequencing (NGS) for the most accurate analysis. The allele frequencies of the all gene mutations were improved by 1.2 times in cells sorted via DEPArray (tumor suppressor genes, 1.3–10.1 times; oncogenes, 1.3–2.6 times). We identified 16 novel mutations using the sequencing from sorted cells via DEPArray technology, compared to detecting 4 novel mutation by the sequencing from unsorted cells. Using this analysis, we also revealed that five genes (*TP53*, *EGFR*, *PTEN*, *RB1*, *KRAS*, and *CTNNB1*) were somatically mutated in multiple homogeneous lung adenocarcinomas. Together, we sorted pure tumor cells from 22 FFPE lung adenocarcinomas by DEPArray technology and identified 16 novel somatic mutations. We also established the precise genomic landscape for more accurate diagnosis in 22 lung adenocarcinomas with mutations detected in pure tumor cells. The results obtained in this study could offer new avenues for the treatment and the diagnosis of squamous cell lung cancers.

**Electronic supplementary material:**

The online version of this article (10.1007/s13353-018-0439-4) contains supplementary material, which is available to authorized users.

## Introduction

Formalin-fixed, paraffin-embedded (FFPE) tissues are used for diagnostic purposes in patients with cancer because FFPE tissues are well-stained immunohistochemically and are storable at room temperature which is a convenient and cost-effective environment (Greytak et al. 2015). Next-generation sequencing (NGS) technology through FFPE tissue has also been attempted to use as a valuable tool for cancer genetic diagnostic purposes (Einaga et al. 2017; Ying 2016). However, there is a huge obstacle in obtaining the accurate NGS data from FFPE tissue, which is difficulty in identifying the somatic and tumor-specific variants in the FFPE tissue due to sequencing artifacts, the lack of normal samples, and heterogeneities in FFPE tissue (Bernstein et al. 2002; Do et al. 2013; Wong et al. 1998). Therefore, NGS data from FFPE tissue is insufficient for assessing the risk of cancer (Petersen et al. 2016). To date, a traditional method such as Sanger sequencing of blood, saliva, and buccal smear has been used to diagnose cancer. The hematoxylin and eosin (H&E) staining slide is reviewed by a pathologist (Snow et al. 2014). However, recent studies have shown that pure tumor cells and pure stromal cell are sorted from blood cells and live cell lines through Di-Electro-Phoretic Array system (DEPArray system) based on the electro-kinetic principle (Fabbri et al. 2013; Fuchs et al. 2006). Additionally, this technology enables the pure tumor cells be sorted from small clinical samples and samples with low tumor cellularity such as FFPE samples (Bolognesi et al. 2016) and can be an efficient research method to avoid bias from heterogeneity of FFPE samples of adenocarcinoma which is the most common type of lung cancer (Calvayrac et al. 2017; Dunne et al. 2016). Although many laboratories have researched for lung adenocarcinoma, most of them have stored the FFPE samples due to difficulty in collecting fresh lung adenocarcinoma tissues and FFPE is the standard method for preserving the most archived pathological specimens for the long-term (Lin et al. 2009). Therefore, development of a new technology is needed for analyzing greater quality of examination to make a more accurate diagnosis of lung cancer in FFPE samples. Here, we performed pure tumor cell isolation from FFPE samples via DEPArray technology and demonstrated more precise genetic analysis using genetic variants from the sorted pure cells.

## Materials and methods

### Information of 22 FFPE lung adenocarcinoma samples

FFPE lung adenocarcinomas were obtained from Korean patients of Seoul National University Hospital in South Korea. The storage time was between 12 and 61 days. Twenty-two FFPE tissue sections (50 μm thickness) were obtained from lung adenocarcinoma tissue block using a standard microtome. After dissociation, the number of the total cells was between 39,000 and 675,000 (Supplementary Table 1). After sorting process via DEPArray system (Silicon Biosystems, Bologna, ITALY), pure tumor cells (100–300), pure stromal cells (100–300), and other minority putative tumor cells (50–90) were isolated from the dissociated cells from 22 FFPE lung adenocarcinomas (Supplementary Fig. S1).

### Cell isolation from FFPE samples

FFPE tissue sections (50 μm thickness) were washed with 10 ml of 100% xylene for 10 min at room temperature. After three times washing with xylene, the samples were rehydrated with 100% ethanol, 70% ethanol, 50% ethanol, and Milli-Q water. After the deparaffinization processes, samples were kept with heat-induced antigen retrieval (HIAR) solution (10 mM sodium citrate buffer) for 5 min at room temperature and for 1 h at 80 °C. Then, the samples were cooled down for 20 min at room temperature and washed with 10 ml of RPMI 1640 (Gibco) at room temperature. After the processes, the samples were dissociated with dissociation buffer (0.1% collagenase Ia (Sigma), 0.1% dispase (Life tech), RPMI), and then filtered with 100-μm mesh nylon filter into 15-ml tube. The samples were washed with ice-cold PBATw (0.05% tween 20, PBS, 1% BSA).

After FFPE tissue dissociation, 5 × 10^5^ cells were stained with anti-keratin MNF116 (IgG1) (DAKO) and anti-keratin AE1/AE3 (IgG1) (Millipore-Chemicon) at room temperature. After first antibody staining, the samples were washed with ice-cold PBATw, and Alexa Fluor 488 goat anti-mouse IgG1 and Alexa Fluor 647 goat anti-mouse IgG2a were used for secondary antibody staining. For DAPI staining, the samples were stained with DNA staining solution (10 μM DAPI (sigma), PBATw) for 30 min at 37 °C.

For sorting process, 5000~10,000 stained cells were loaded into DEPArray system and were analyzed to isolate pure cells via the software of DEPArray system. Keratin−/Vimentin+ population, Keratin+/Vimentin− population, and Keratin+/Vimentin+ population were gated and sorted by DEPArray system for pure cells (Keratin−/Vimentin+ population, pure stromal cells; Keratin+/Vimentin− population, pure tumor cells; Keratin+/Vimentin+ population, other minority putative tumor cells).

### Targeted sequencing

The next-generation sequencings were performed by using the Ion AmpliSeq Cancer Panel v2 (Life Technologies) that can detect 2800 COSMIC mutations of 50 oncogenes and tumor suppressor genes.

The Ion Torrent Libraries were prepared with the Ion Ampliseq library kit 2.0 (Life Technologies), quantified by the Qubit dsDNA HS Assay kit (Life Technologies), and the sizes of libraries were analyzed with Agilent Bioanalyzer 2100 system. The enrichment process for libraries was performed using the Ion Personal Genome Machine (PGM) Template OT2 200 Template Kit and the Ion One Touch 2 instrument. The prepared libraries were pooled on a 316™ Chip (Life Technologies) per six libraries and sequenced the Ion Torrent Ion Personal Genome Machine (PGM) system™ (Life Technologies). All procedures for targeted sequencing for the Ion AmpliSeq Cancer Panel v2 (Life Technologies) were conducted according to the manufacturer’s protocol.

### Data analysis

The sequenced data were processed with Torrent Suite 4.4.3 and were aligned to the *Homo sapiens* hg19 reference genome. Variants were generated by the Torrent Variant Caller and annotated by Annovar (Wang et al. 2010) that used databases such as dbSNP138 (Smigielski et al. 2000), clinvar (Landrum et al. 2016), 1000 genomes, polypen2, the exome aggregation consortium (EXAC), and sorting tolerant from intolerant (SIFT) algorithm (Ng and Henikoff 2003). The variants were visually validated by using The Integrative Genomics Viewer (IGV) (Robinson et al. 2017; Thorvaldsdottir et al. 2013). False-positive variants were excluded because they were found in misalignments.

### Somatic mutation and germline mutation analysis

Somatic mutations and germline mutations were analyzed with variants called in sorted pure stromal cells and variants called in pure sorted tumor cells.

### Pathway analysis

Pathway analysis was performed for genes having mutations in each tumor utilizing Kyoto Encyclopedia of Genes and Genomes (KEGG) (Kanehisa 2002). Mutational spectra for mutated genes were screened on published papers and were manually searched the KEGG pathway database.

## Results

### Summary of workflow

**Fig. 1:**
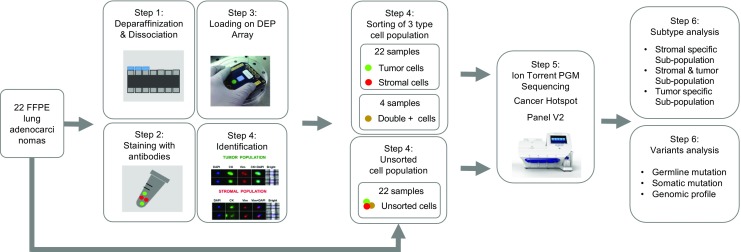
Experimental workflow. This flow chart provides brief experimental step including FFPE sampling, sequencing processing, and variants analysis

It is an important factor for accurate cancer diagnosis and precise treatments to detect specific variants in FFPE samples (Mafficini et al. 2014a). Attempts have been made to identify the variants in FFPE samples, but there were several obstacles because of technical issues including the heterogeneity of FFPE tissues and sequence artifacts in DNA from FFPE (Adank et al. 2006). We sorted pure stromal cells and pure tumor cells from 22 lung adenocarcinoma formalin-fixed paraffin-embedded (FFPE) blocks via DEPArray system to perform a more precise genetic variant analysis of FFPE pure tumor tissue. We respectively found variants from pure stromal cells and pure tumor cells collected from each of the 22 FFPE samples via DEPArray technology to improve homogeneity of tumor cells and to identify somatic mutations. Pure double-positive cells (keratin+/vimentin+) were also recovered from four FFPE samples to analyze cells excluding stromal cells and tumor cells in FFPE sample. We extracted DNA from sorted cells and unsorted cells. The DNA samples were sequenced with cancer hotspot panels (Life Technologies, Waltham, MA USA) on Ion Torrent PGM (Life Technologies, Waltham, MA USA). Functional effect of the variants was predicted by polypen2 and SIFT. The results for variants were analyzed to explore the heterogeneity and characteristics of FFPE samples (Fig. 1).

### Heterogeneity of FFPE samples

**Fig. 2:**
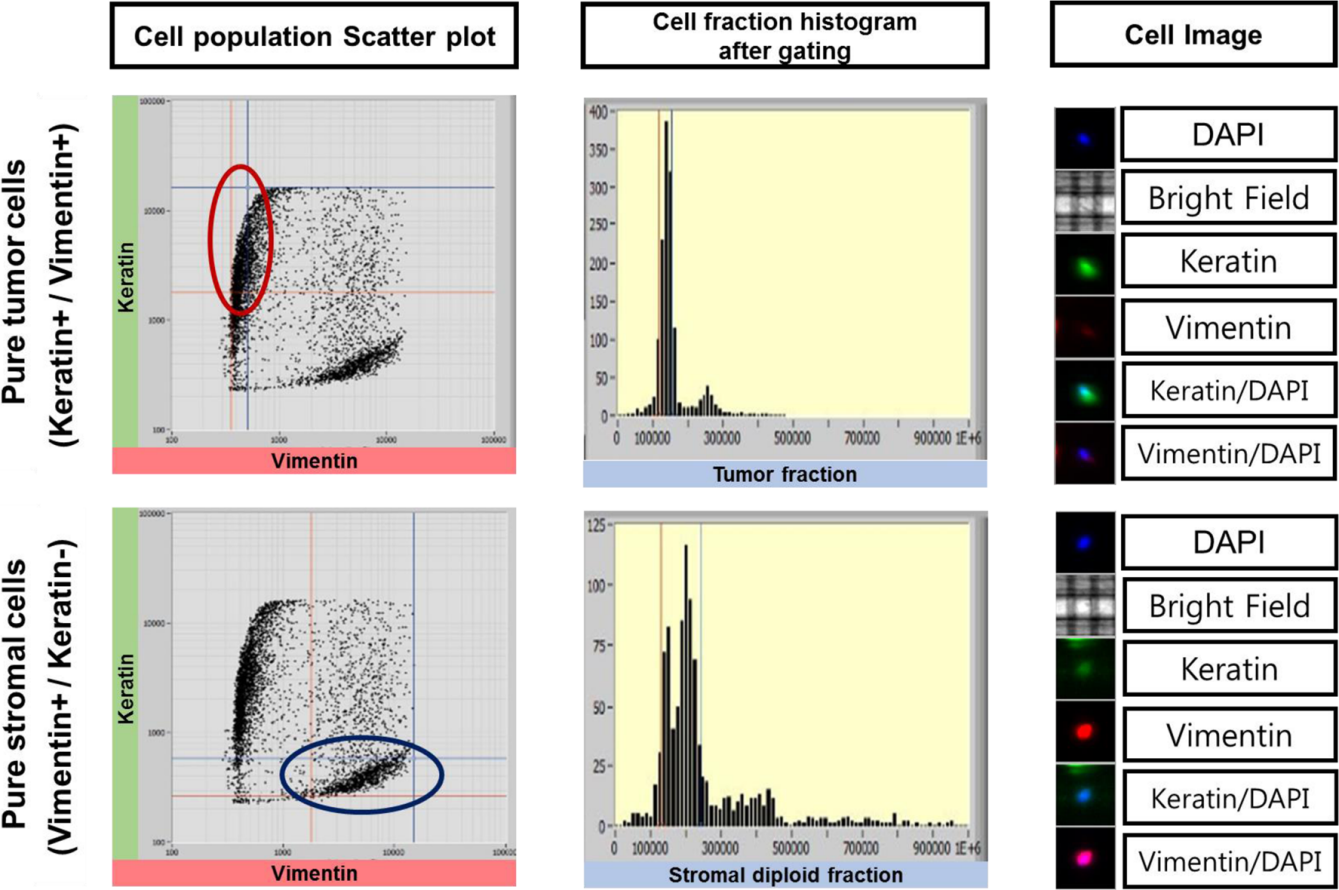
Cell analysis and pure cell sorting via DEParray technology. Cell populations in FFPE sample (left), cell populations after gating (middle), and stained cell images (right), which are analyzed by DEPArray technology for cell sorting, are plotted

**Fig. 3:**
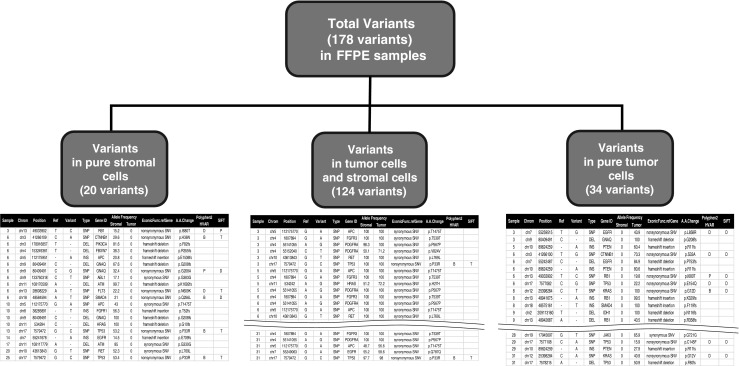
Heterogeneity in FFPE samples. Stromal-specific variants, tumor and stromal sharing variants, and tumor-specific variants are shown in total variants of unsorted FFPE samples

Although FFPE samples were designed to diagnose tumors, FFPE blocks included non-tumor cells such as stromal cells. It is difficult to extract pure tumor DNA from FFPE samples. Heterogeneity of FFPE has been detected in FFPE samples previously. Significant differences in variants were displayed even in the same tumor FFPE samples (Mafficini et al. 2014). Enhancement of homogeneity in FFPE tumor samples is very important for developing targeted gene therapies. To improve homogeneity of tumor cells and to detect tumor variants for a more accurate cancer diagnosis and research, we analyzed cell populations in FFPE lung adenocarcinoma and sorted the stromal cell population (Keratin−/Vimentin+), the tumor cell population (Keratin+/Vimentin−), and the double-positive cell population (Keratin+/Vimentin+) from 22 FFPE lung adenocarcinoma samples via The DEPArray System (Fig. 2 and Supplementary Fig. S1). We analyzed variants in sorted pure tumor cells and sorted pure stromal cells to investigate the heterogeneity in FFPE samples and discovered 34 tumor-specific somatic variants in sorted tumor samples. We found that different mutation patterns were shown in each subgroup, sorted from FFPE samples (Fig. 3 and Supplementary Table 2A–C). This suggests that several subtypes besides tumor cells are in unsorted FFPE samples and mislead the research and diagnosis of lung adenocarcinoma.

### Improved detection of variants in sorted cells from FFPE samples

**Fig. 4:**
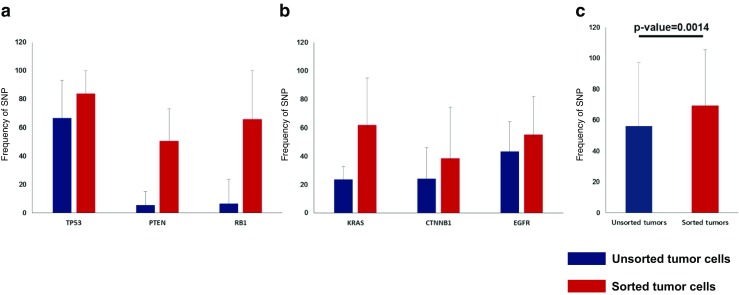
Sensitivities for variants detection between sorted cells sequencing and unsorted cell sequencing. **a** Sensitivities for SNP detection in unsorted tumor cells (blue) and in sorted tumor cells (red) are shown across three tumor suppressor genes. **b** Sensitivities for SNP detection in unsorted tumor cells (blue) and sorted tumor cells (red) are shown across three oncogenes. **c** Sensitivities for total SNP detection are shown across unsorted tumor cells and sorted tumor cells

To improve the accuracy of detection of tumor variants, we isolated 100~300 pure tumor cells, and sorted pure tumor cells were sequenced for detecting variants in cancer hot spot regions. Using DEPArray technology and NGS sequencing, we identified 20 stromal-specific variants, which would cause bias for accurate diagnosis, in sequencing data of unsorted FFPE samples. We also found 34 tumor-specific variants detected in only sorted tumor cells (Fig. 3). The allele frequencies of sorted tumor cell variants were increased by 1.3–10.1 times in three tumor suppressor genes such as TP53, PTEN, and RB1 (Fig. 4a) and by 1.3–2.6 times in three oncogenes such as KRAS, CTNNB1, and EGFR (Fig. 4b). Allele frequencies of the all gene mutations were increased by 1.2 times in sorted cells (Fig. 4c). These suggests that the more accurate mutation information was detected through DEPArray technology and NGS sequencing.

### Novel mutations detected by sorted cell sequencing and characteristic of somatic mutations in lung adenocarcinomas

**Fig. 5:**
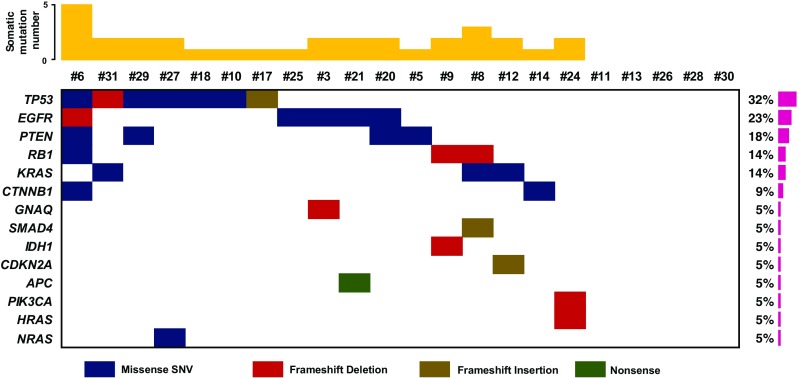
Mutation profiling . Name of significantly mutated genes (left), distribution of mutations across 22 lung adenocarcinomas (middle), and frequency of significantly mutated genes (right) are plotted (bottom). Somatic mutation numbers are shown across patients (top)

**Fig. 6:**
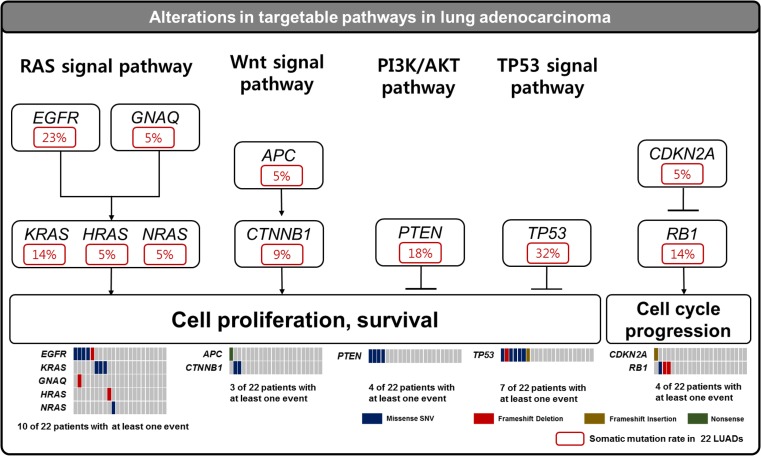
Somatically altered pathways in FFPE lung adenocarcinomas. Somatically altered pathways are plotted with somatic mutations in cell cycle progression and cell proliferation and survival-related pathways

Thirty-four somatic mutations across 16 genes were identified in 22 pure sorted lung adenocarcinomas. Sixteen mutations of 34 somatic mutations were novel and unreported in dbSNP, COSMIC, EXAC, and 1000 genome database (Table 1). We found four novel mutations by the sequencing of unsorted cells, but revealed 12 more novel mutation by the sequencing of sorted tumor cells (Supplementary Fig. S2). One hundred twenty-six germline mutations were also discovered, and three mutations of them were unpublished in dbSNP, COSMIC, EXAC, and 1000 genome database (Supplementary Table 3). Especially RB1 (p.I680T) of 16 newly identified somatic mutations were evaluated to deleterious in PROVEAN and SIFT (Table 1). Based on somatic mutations detected by sorted cell sequencing, *TP53*, *EGFR*, *PTEN*, *RB1*, *KRAS*, *CTNNB1*, *GNAQ*, *SMAD4*, *IDH1*, *CDKN2A*, *APC*, *PIK3CA*, *HRAS*, and *NRAS* were observed significantly in 22 lung adenocarcinomas (Fig. 5). Using this mutation profile, we also revealed five core somatically mutated pathways: RAS signaling pathway (ten cases, 45%), WNT signaling pathway (three cases, 14%), PIK3K/AKT signaling pathway (four cases, 18%), TP53 signaling pathway (seven cases, 32%), and cell cycle progression pathway(four cases, 18%) (Fig. 6).

**Table 1:** Somatic mutations identified using sorted cell sequencing

Sample	Gene	Position	Ref	Alt	Type of alteration	NM number	AA change	Provean prediction	SIFT prediction	dbSNP ID	COSMIC ID	EXAC	1000 Genome	Unsorted cell sequencing	Sorted cell sequencing
#3	*EGFR*	chr7:55259515	T	G	missense	NM_005228	p.L858R	Deleterious	Deleterious	rs121434568	COSM6224			Detected	Detected
#3	*GNAQ*	chr9:80409491	C	–	frameshift deletion	NM_002072	p.G208 fs							Not detected	Detected
#5	*PTEN*	chr10:89624259	–	A	frameshift insertion	NM_000314	p.R11fs							Not detected	Detected
#6	*CTNNB1*	chr3:41266100	T	G	missense	NM_001098209	p.S33A	Deleterious	Deleterious		COSM5683			Detected	Detected
#6	*EGFR*	chr7:55242487	C	–	frameshift deletion	NM_005228	p.P753fs							Detected	Detected
#6	*PTEN*	chr10:89624259	–	A	frameshift insertion	NM_000314	p.R11fs							Not detected	Detected
#6	*RB1*	chr13:49033902	T	C	missense	NM_000321	p.I680T	Deleterious	Deleterious					Not detected	Detected
#6	*TP53*	chr17:7577082	C	G	missense	NM_001126115	p.E154Q	Deleterious	Deleterious		COSM1480057			Not detected	Detected
#8	*KRAS*	chr12:25398284	C	T	missense	NM_004985	p.G12D	Deleterious	Deleterious	rs121913529	COSM521	Reported		Detected	Detected
#8	*RB1*	chr13:48941675	–	A	frameshift insertion	NM_000321	p.K329 fs							Not detected	Detected
#8	*SMAD4*	chr18:48575161	–	T	frameshift insertion	NM_005359	p.F119 fs							Not detected	Detected
#9	*IDH1*	chr2:209113160	T	–	frameshift deletion	NM_001282386	p.N116 fs							Not detected	Detected
#9	*RB1*	chr13:48942687	A	–	frameshift deletion	NM_000321	p.R358fs							Not detected	Detected
#10	*TP53*	chr17:7577534	C	A	missense	NM_001126115	p.R117S	Deleterious	Deleterious	rs28934571	COSM131478			Detected	Detected
#12	*KRAS*	chr12:25398284	C	A	missense	NM_004985	p.G12 V	Deleterious	Deleterious	rs121913529	COSM1140133			Detected	Detected
#12	*CDKN2A*	chr9:21971125	–	A	frameshift insertion	NM_000077	p.L78 fs							Not detected	Detected
#14	*CTNNB1*	chr3:41266101	C	G	missense	NM_001098209	p.S33C	Deleterious	Deleterious	rs121913400	COSM5677			Detected	Detected
#17	*TP53*	chr17:7578398	–	G	frameshift insertion	NM_001126115	p.H46fs							Not detected	Detected
#18	*TP53*	chr17:7577538	C	T	missense	NM_001126115	p.R116Q	Deleterious	Deleterious	rs11540652	COSM99021	Reported		Detected	Detected
#20	*EGFR*	chr7:55259515	T	G	missense	NM_005228	p.L858R	Deleterious	Deleterious	rs121434568	COSM6224			Detected	Detected
#20	*PTEN*	chr10:89692866	A	C	missense	NM_000314	p.N117 T	Neutral	Tolerated					Detected	Detected
#21	*KIT*	chr4:55599301	T	C	silent	NM_000222	p.C809C							Detected	Detected
#21	*APC*	chr5:112175423	C	T	nonsense	NM_001127511	p.Q1360X			rs121913329	COSM18862			Detected	Detected
#21	*EGFR*	chr7:55259515	T	G	missense	NM_005228	p.L858R	Deleterious	Deleterious	rs121434568	COSM6224			Detected	Detected
#24	*PIK3CA*	chr3:178916857	T	–	frameshift deletion	NM_006218	p.F82 fs			rs141098973				Detected	Detected
#24	*HRAS*	chr11:534294	C	–	frameshift deletion	NM_001130442	p.G10 fs							Not detected	Detected
#25	*EGFR*	chr7:55259515	T	G	missense	NM_005228	p.L858R	Deleterious	Deleterious	rs121434568	COSM6224			Detected	Detected
#27	*NRAS*	chr1:115256529	T	C	missense	NM_002524	p.Q61R	Deleterious	Deleterious	rs11554290	COSM584			Detected	Detected
#27	*TP53*	chr17:7578203	C	A	missense	NM_001126115	p.V84 L	Deleterious	Deleterious		COSM1386669			Detected	Detected
#28	*JAK3*	chr19:17945697	G	T	silent	NM_000215	p.G721G							Detected	Detected
#29	*TP53*	chr17:7577108	C	A	missense	NM_001126115	p.C145F	Deleterious	Deleterious		COSM562338	Reported		Detected	Detected
#29	*PTEN*	chr10:89624259	–	A	frameshift insertion	NM_000314	p.R11fs							Not detected	Detected
#31	*KRAS*	chr12:25398284	C	A	missense	NM_004985	p.G12 V	Deleterious	Deleterious	rs121913529	COSM1140133			Detected	Detected
#31	*TP53*	chr17:7578215	A	–	frameshift deletion	NM_001126115	p.F80 fs				COSM44358			Not detected	Detected

Ref seq, reference sequence; Alt seq, alternate sequence;AA, amino acid

## Discussion

Nowadays, we have incorporated next-generation sequencing (NGS) technology from a research environment into clinical practice (Shen et al. 2015). Accuracy and precision of NGS technology are required for making a clinical diagnosis (Pinho 2017). To identify the causes and to develop strategies for prevention, diagnosis, and treatment of lung adenocarcinoma, it is very important to classify somatic variants developed in cancer based on mutagen and germline variants passed from a parent to a child and able to be inherited cancer. We identified 34 somatic mutations across 16 genes and 126 germline mutations across 17 genes including 10 germline mutations unreported in dbSNP and COSMIC. Most of germline mutations (88%) were also detected by traditional sequencing method without cell sorting. Ninety-three out of 126 germline mutations were silent SNVs, and only three out of 126 germline mutations were unenrolled in dbSNP, COSMIC, EXAC, and 1000 genome database (Supplementary Table 3). However, in the case of somatic mutation analysis, we discovered 20 somatic mutations including 4 novel somatic mutations by the sequencing of unsorted cells, and 14 more somatic mutation including 12 novel mutations by the sequencing of sorted tumor cells (Supplementary Fig. S2b). These imply that sorted cell sequencing is more accurate for somatic mutation diagnosis. These imply that germline mutations were detected fully by traditional next-generation sequencing, but tumor-specific somatic mutation, which is significant factor for cancer diagnostics, was observed more sensitively by sequencing from sorted pure tumor cells.

We found that there are epithelial-to-mesenchymal transition (EMT) sub-populations in FFPE samples. Epithelial mesenchymal transition causes embryonic development and cancer progression. Epithelial-to-mesenchymal transition (EMT), which indicates the conversion of epithelial cells to migratory mesenchymal cells, has been shown by intermediate keratin/vimentin expression ratios (Polioudaki et al. 2015), and we sorted stromal and tumor cells with vimentin antibody and keratin antibody. Further study with sorted cells as keratin/vimentin expression ratios is needed for assessing EMT characteristics in lung adenocarcinoma.

As the results of current study, DEPArray system is a very useful tool to identify mutations from small amount of tumor cells, to avoid false-positive mutation and to find the most accurate mutations from FFPE tumor samples. However, the system also has a limitation that the system is difficult to handle large number of cells from large volume of cancers because of sorting time and the expenses.

In conclusion, we successfully established precise mutational analysis of lung adenocarcinoma and identified 16 unreported somatic mutation and 10 germline mutations in block using sorted technology-applied NGS method. Newly detected mutations and our accurate mutational profiling, using sorted technology-applied NGS method, will be suitable to research main causes of adenocarcinoma and critical factors for precision medicine of lung adenocarcinoma. Additionally, characteristics of all variants were considered because somatic variants were a feature of cancer and germline variants are a cause of heritable diseases.

## Electronic supplementary material

Supplementary Figure S1 The numbers of pure cells, which are sorted by DEParray technology, are displayed. (a) Sorted tumor cells, (b) Sorted stromal cells, (c) Sorted other minority putative tumor cells. (PPTX 70 kb)

Supplementary Table 1 Sample list (XLSX 11 kb)

Supplementary Table 2 A. Variants in sorted pure stromal cells. B. Variants in sorted pure stromal cells and sorted pure tumor cells C. Variants in sorted pure tumor cells (XLSX 24 kb)

Supplementary Table 3 Germline mutations identified using sorted cell sequencing (XLSX 18 kb)
