# Supplementary material for: Identification of novel mutations in FFPE lung adenocarcinomas using DEPArray sorting technology and next-generation sequencing
Source: J Appl Genet. 2018 Mar 10;59(3):269–77. doi: 10.1007/s13353-018-0439-4 (PMC6060994; doi:10.1007/s13353-018-0439-4)
Supplement: Supplementary file 1 — The numbers of pure cells, which are sorted by DEParray technology, are displayed. (a) Sorted tumor cells, (b) Sorted stromal cells, (c) Sorted other minority putative tumor cells. (PPTX 70 kb) [file 13353_2018_439_MOESM1_ESM.pptx]

## Slide 1
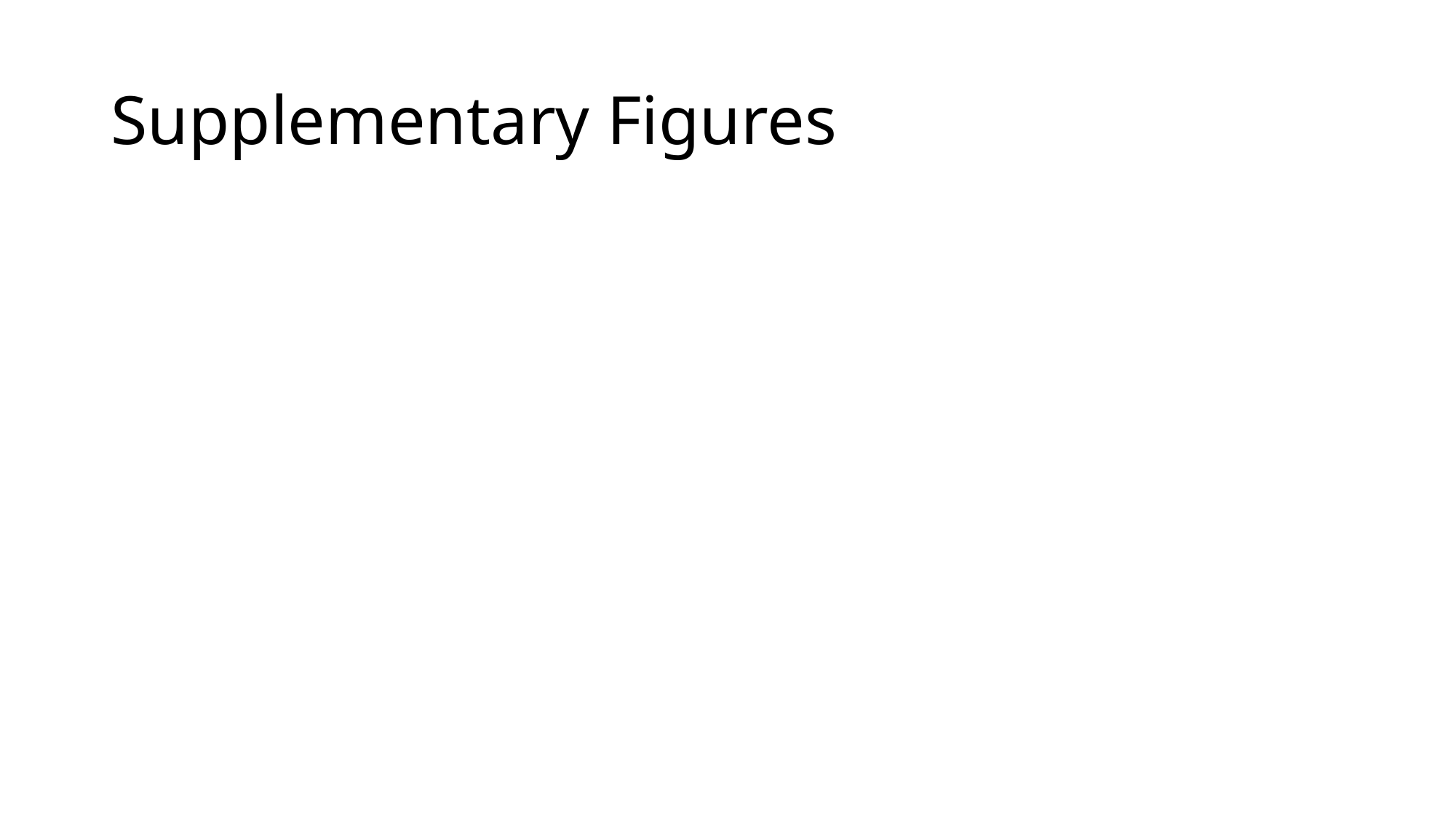

# Supplementary Figures

## Slide 2
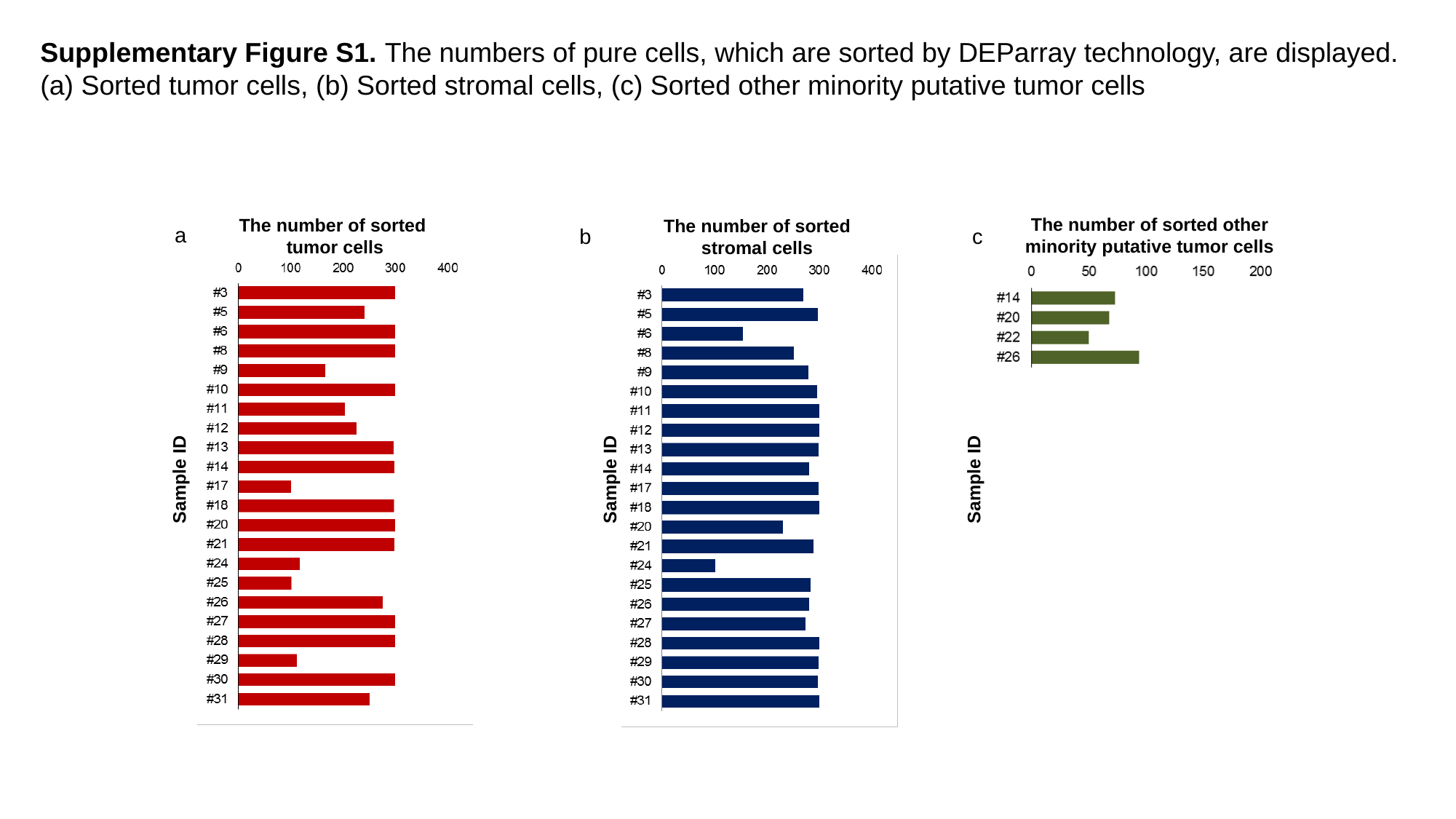

Supplementary Figure S1. The numbers of pure cells, which are sorted by DEParray technology, are displayed. (a) Sorted tumor cells, (b) Sorted stromal cells, (c) Sorted other minority putative tumor cells
a
b
c
The number of sorted other minority putative tumor cells
The number of sorted
tumor cells
The number of sorted stromal cells
Sample ID
Sample ID
Sample ID

## Slide 3
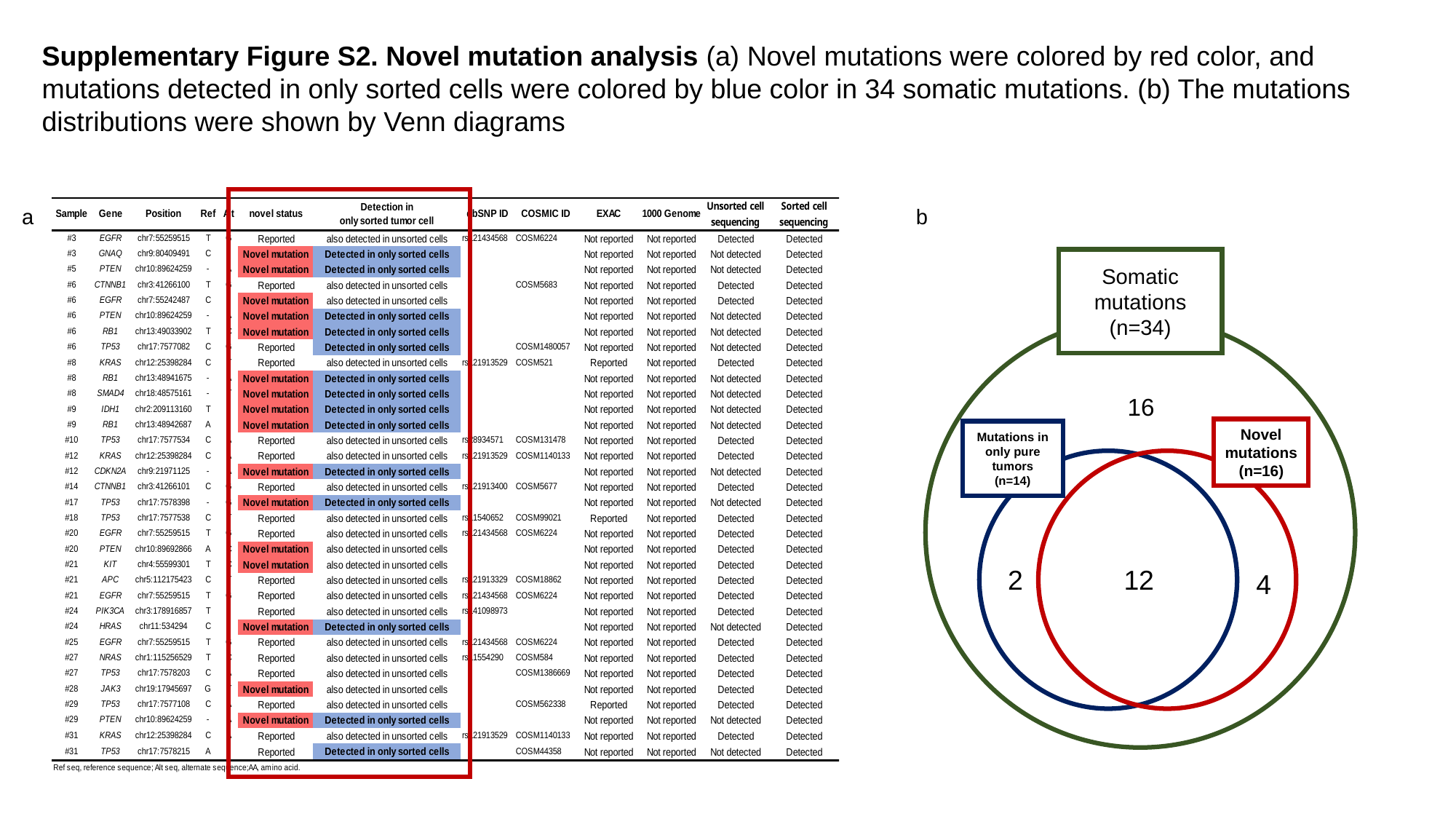

Supplementary Figure S2. Novel mutation analysis (a) Novel mutations were colored by red color, and mutations detected in only sorted cells were colored by blue color in 34 somatic mutations. (b) The mutations distributions were shown by Venn diagrams
b
a
Somatic mutations
(n=34)
16
Novel
mutations
(n=16)
Mutations in only pure tumors
(n=14)
2
12
4
